# Reducing Companion Animal Abandonment During Disaster-Driven Relocation: A Four-Year Study in Maceió, Brazil

**DOI:** 10.3390/ani16101478

**Published:** 2026-05-12

**Authors:** Keityane de Oliveira e Silva, Helena Emília Oliveira Teodosio, Juliana de Oliveira Bernardo, Sharacely de Souza Farias, Pierre Barnabé Escodro

**Affiliations:** Research and Extension Group on Equines and Integrative Health, Federal University of Alagoas, Viçosa 57700-000, AL, Brazil; helena.teodosio@ceca.ufal.br (H.E.O.T.); juliana.bernardo@vicosa.ufal.br (J.d.O.B.); shrfarias@gmail.com (S.d.S.F.); pierre.escodro@vicosa.ufal.br (P.B.E.)

**Keywords:** mining, animal abandonment, responsible ownership, relocation

## Abstract

In March 2018, seismic events associated with rock salt mining in Maceió, northeastern Brazil, led to the emergency relocation of families from risk areas, resulting in increased companion animal abandonment. This study assessed the association between systematic monitoring and environmental education and the reduction in abandonment during these relocation processes. Between March 2018 and September 2020, 567 animals were recorded in affected households, of which only 245 (43.2%) were relocated with their guardians. In response, the Integra Animal Project was implemented, integrating environmental education, continuous monitoring, sanitary management, and population control. By December 2024, 2559 households and 6673 animals had been monitored. A substantial reduction in abandonment and escape rates was observed over time, with abandonment decreasing from 56.8% to 5.45%. Cats showed significantly higher escape rates than dogs (chi-square test). These findings suggest that integrated strategies combining monitoring and environmental education are associated with improved animal retention during disaster-driven relocation, supporting their relevance for animal welfare, public health, and One Health approaches.

## Simple Summary

The forced displacement of families from areas affected by land subsidence in Maceió was associated with an increase in the number of companion animal abandonments, likely reflecting challenges faced by guardians in relocating with their animals. This study assessed whether monitoring relocations and implementing educational actions focused on animal care helped reduce this problem. Household moves were monitored, with guidance provided to guardians, support during animal transport, provision of vaccination, parasite control, sterilization, and training of the teams involved. Between 2020 and 2024, 6673 animals were monitored during this process. The results showed a marked reduction in abandonment, decreasing from 56.8% of animals recorded at baseline (*n* = 567) to approximately 5% after the implementation of these actions. The study demonstrates that including animals in relocation planning and providing support to guardians are simple and effective measures to protect animals, reduce public health risks, and minimize environmental impacts. These strategies can also be applied in other similar situations.

## 1. Introduction

The relationship between humans and companion animals has undergone significant transformations over recent decades, with pets increasingly being regarded as family members. This shift has important implications for animal welfare, public health, and disaster preparedness, particularly in situations where human populations are exposed to environmental or socio-environmental disruptions. In disaster scenarios, the bond between guardians and their animals can directly influence evacuation decisions, risk exposure, and post-displacement outcomes [1,2].

Despite their social relevance, companion animals are frequently overlooked in disaster planning and response strategies. Consequently, events such as floods, landslides, and forced relocations are often associated with increased rates of animal abandonment, loss, and mortality. In many cases, guardians are unprepared to ensure animal care under emergency conditions, contributing to the disruption of the human–animal bond [3,4].

Abandoned animals are frequently exposed to adverse situations that compromise their welfare, including food and water scarcity, exposure to extreme environmental conditions, increased risk of infectious and parasitic diseases, injuries caused by traffic accidents or aggression, and chronic stress. These conditions negatively affect both physical health and behavioral welfare, reducing survival rates and increasing suffering [5,6].

At the beginning of 2018, residents of five neighborhoods in the city of Maceió, capital of Alagoas, Brazil, reported structural cracks in buildings and ground depressions. Concern intensified after a magnitude 2.4 earthquake on March 3 of the same year [7]. More than 14,000 buildings were subsequently classified as being in risk areas, leading to the relocation of approximately 55,000 people. Investigations revealed that decades of rock salt extraction caused instability in underground cavities, resulting in land subsidence and structural damage, as well as the suspension of mining activities in 2019 [8,9,10].

From an environmental perspective, subsidence has severe impacts on fauna and flora, as soil collapse, crater formation, and environmental contamination compromise ecosystems and lead to biodiversity loss [11,12,13]. The forced displacement of human populations further contributes to animal abandonment, increasing the number of free-roaming animals and intensifying interactions between domestic animals and wildlife. These dynamics may facilitate zoonotic disease transmission, promote ecological imbalance, and impact local biodiversity, reinforcing the need for integrated One Health strategies [14,15,16,17,18].

In Brazil, companion animal population dynamics are influenced by cultural, socioeconomic, and regional factors. Although dogs are generally more prevalent, some regions report a higher proportion of cats, often associated with perceptions of greater independence and lower maintenance requirements [19,20]. This perception may lead to underestimation of risks during relocation, potentially increasing vulnerability to abandonment. However, it remains unclear whether this pattern reflects broader national trends or specific local conditions [21,22].

Animal abandonment in crisis situations is a multifactorial issue with significant implications for animal welfare and public health, particularly in Latin America [23,24,25,26]. In this context, baseline data from the Mutange neighborhood, the first area to be evacuated, illustrate the magnitude of the problem: of the 567 animals registered prior to relocation, only 245 were successfully relocated, indicating that 56.8% were abandoned or lost during the process [15]. This scenario highlights the limitations of unstructured relocation processes and reinforces the need for coordinated strategies to prevent abandonment during forced displacement.

Within this framework, integrated strategies combining environmental education and continuous monitoring have been proposed as approaches to mitigate abandonment and improve animal welfare during displacement processes [6,27,28,29]. Environmental education may support behavioral change by promoting responsible ownership, while monitoring enables the identification of risk situations and timely interventions [14].

Evidence from community-based interventions also suggests that educational and monitoring actions can contribute to reducing abandonment and improving animal welfare outcomes in socio-environmental crisis contexts [30]. However, despite the recognized importance of these strategies, there is still a lack of longitudinal, field-based studies evaluating their effectiveness in real-world disaster-driven relocation scenarios [4]. In addition, limited attention has been given to multispecies families and the specific challenges associated with maintaining the human–animal bond during forced displacement [2], with most existing studies focusing on short-term or emergency responses.

Therefore, this study contributes to the literature by providing longitudinal, field-based evidence on the effectiveness of integrated strategies, combining environmental education, monitoring, and population control strategies, in reducing companion animal abandonment during disaster-driven relocation processes.

To address this gap and provide empirical evidence on the effectiveness of integrated strategies in real-world contexts, an intervention hereafter referred to as the Integra Animal Project was implemented through a collaboration between public institutions, academia, and the private sector. The initiative was structured around three main pillars—Education, Public Health, and Animal Welfare—and included actions such as animal sheltering, sterilization programs, environmental education, and continuous monitoring. This intervention constituted the operational basis for evaluating the strategies analyzed in the present study.

Therefore, this study aims to evaluate the impact of monitoring and environmental education strategies on reducing companion animal abandonment during disaster-driven relocation over a four-year period in Maceió, Brazil.

## 2. Materials and Methods

This is a longitudinal observational study based on data collected between October 2020 and December 2024 as part of the Integra Animal Project, an extension and research initiative developed by the Federal University of Alagoas (UFAL), Brazil. The project was approved by the Animal Use Ethics Committee (CEUA/UFAL; protocol no. 33/2020) and registered at the university’s Extension Office (PJ 174–2020).

The study followed a real-world intervention framework, combining animal monitoring and environmental education strategies implemented during a disaster-driven relocation process affecting multiple neighborhoods in Maceió, Alagoas, Brazil.

### 2.1. Study Area and Population

The study was conducted in neighborhoods affected by ground subsidence caused by rock salt extraction (Pinheiro, Bebedouro, Mutange, Bom Parto, and Farol), which resulted in the forced relocation of thousands of residents.

The study population included dogs and cats belonging to multispecies households undergoing relocation, as well as free-roaming animals identified within the affected areas during the study period.

### 2.2. Definition of Animal Status

For the purposes of this study, animals were classified into the following categories:

- Abandoned animals: animals without active guardianship, identified as having been left behind during relocation or intentionally discarded in affected areas. Classification was based on field verification (absence in the new residence), lack of owner identification, and/or reports from community members.
- Escaped animals: animals reported by guardians as unintentionally lost during the relocation process, with evidence of prior ownership and attempts at recovery.
- Owned animals: animals that remained under the care of their guardians during and after relocation.

To minimize misclassification, cases were cross-checked using field visits, guardian reports, and project records.

### 2.3. Monitoring Procedures

Continuous monitoring was conducted throughout the study period by trained personnel affiliated with the Integra Animal Project, including veterinarians, students, and extension team members.

For the purposes of this study, continuous monitoring was defined as the systematic and repeated follow-up of households and animals across all stages of the relocation process, including pre-relocation, relocation, and post-relocation phases.

Monitoring involved:

- Pre-relocation visits conducted approximately 48 h in advance
- On-site monitoring during relocation procedures
- Post-relocation follow-up visits to verify animal presence and adaptation
- Periodic field visits to affected neighborhoods and relocation areas
- Household follow-up to verify the presence or absence of animals
- Recording of animal status (maintained, abandoned, escaped, recovered)
- Identification of risk situations (e.g., inadequate care, potential abandonment)

Households were followed longitudinally whenever possible, with repeated observations over time, allowing the identification of temporal trends in abandonment and recovery.

Data were recorded using standardized forms and later compiled into a centralized database for analysis.

Not all animals received all interventions, as their implementation depended on factors such as guardian availability, logistical constraints, and animal eligibility (e.g., age or clinical condition for sterilization). Monitoring and environmental education activities were broadly applied across the study population, whereas veterinary interventions such as vaccination, treatment, and sterilization were performed whenever feasible and upon authorization from the responsible guardian, when applicable.

### 2.4. Environmental Education Interventions

Environmental education activities were implemented as a central component of the Integra Animal Project, with the objective of promoting responsible ownership, preventing animal abandonment, and strengthening the human–animal bond during the relocation process.

The interventions were conducted through a combination of school-based programs, community outreach activities, and field-based guidance provided during monitoring visits, allowing continuous engagement with both children and adult guardians.

School-based interventions consisted of structured educational sessions delivered in public schools located in or near the affected areas. These sessions addressed key topics such as animal welfare, responsible ownership, legal aspects of animal protection, sterilization, disease prevention, and the One Health concept. Interactive methodologies were used, including lectures, group discussions, and educational materials adapted to different age groups, aiming to encourage active participation and knowledge dissemination within families and communities.

Community outreach activities included awareness campaigns and direct engagement with residents during field visits. Guardians received guidance on appropriate animal care, the importance of maintaining animals during relocation, and strategies to prevent abandonment. Informational materials were also distributed when available.

In addition, educational actions were integrated with other project activities, such as vaccination campaigns, sterilization programs, and animal rescue efforts. This integrated approach allowed reinforcement of key messages in practical contexts, facilitating behavioral change.

All activities were conducted by trained personnel, including veterinarians, undergraduate students, and extension team members affiliated with the Federal University of Alagoas. The multidisciplinary nature of the team contributed to addressing both technical and social aspects of animal care.

The educational strategy followed a continuous and adaptive approach throughout the study period, with repeated interactions with the same communities whenever possible. Although no formal quantitative assessment of learning outcomes was performed, qualitative observations from field teams indicated increased awareness among guardians and greater adherence to responsible ownership practices.

A detailed description of the structure and implementation of these educational actions has been previously reported (Silva et al., 2025 [30]).

### 2.5. Data Collection

Data collected included:

- Number of animals per household
- Species, age group, and sex
- Animal status (maintained, abandoned, escaped, recovered)
- Access to veterinary care (e.g., vaccination, sterilization, treatment)
- Participation in relocation processes

Whenever applicable, data were obtained directly from guardians. In cases where this was not possible, information was collected through field observation and project records.

To ensure confidentiality, no personally identifiable information from guardians was recorded.

### 2.6. Statistical Analysis

Statistical analyses were performed using Microsoft Excel (Microsoft Corp., Redmond, WA, USA). Descriptive statistics were used to summarize the data, including absolute and relative frequencies.

The association between species and escape occurrence was evaluated using the chi-square test.

Confidence intervals (95% CI) were calculated for selected proportions, including escape rates by species, to provide an estimate of precision.

Temporal trends in abandonment and escape rates were assessed descriptively over the study period, as the observational design and non-uniform data collection over time precluded the use of inferential time-series analyses.

The level of statistical significance was set at *p* < 0.05.

## 3. Results

A total of 2559 households were monitored and resettled during the study period. The number of monitored residences per year was 534 in 2020, 1320 in 2021, 435 in 2022, 236 in 2023, and 34 in 2024. Among the affected neighborhoods, the highest number of relocations was recorded in Bebedouro (971; 38.0%), followed by Pinheiro (576; 22.5%), Farol (574; 22.4%), Bom Parto (383; 15.0%), and Mutange (55; 2.1%).

### 3.1. Relocation and Population Characteristics

A total of 6673 animals were assisted and relocated, with a mean of 2.6 animals per household. The annual distribution included 1340 animals (20.1%) in 2020, 3243 (48.6%) in 2021, 1071 (16.0%) in 2022, 909 (13.6%) in 2023, and 110 (1.6%) in 2024. A decreasing trend in the number of animals assisted per year was observed after 2021, reflecting the progressive completion of relocation processes.

**Figure 1:**
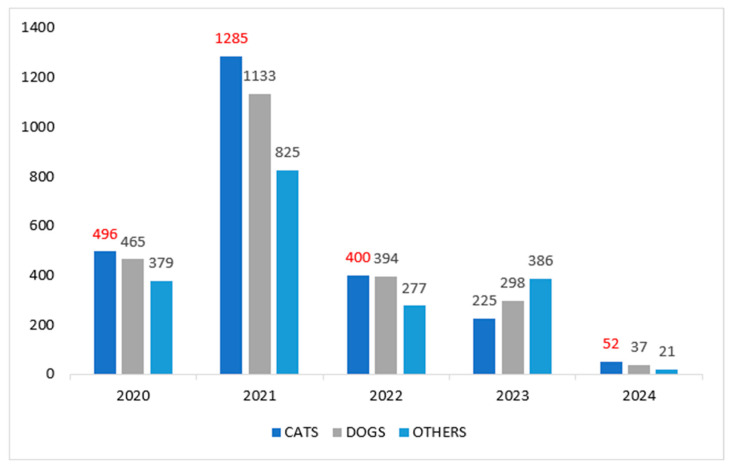
Number of animals assisted by species during relocations in neighborhoods affected by subsidence in Maceió, from October 2020 to December 2024, in the city of Maceió, Alagoas. Integra Animal Project (2024). The source highlighted in red represents the period in which the number of cats assisted was greater than that of other species.

Species distribution included 2531 cats (37.9%), 2254 dogs (33.8%), and 1888 animals of other species (28.3%). Cats predominated across most evaluated periods, representing 37.0% of records in 2020, 39.6% in 2021, 37.3% in 2022, and 47.3% in 2024 (Figure 1).

### 3.2. Relocation Conditions and Management

Most relocations (4192; 78.7%) were conducted by contracted transport companies, while 1136 (21.3%) were performed directly by guardians. Among 244 transport crates evaluated, 89 (36.5%) presented structural damage, and in 151 relocations (3.6%) the crates did not arrive on time.

A total of 583 professionals participated in training activities aimed at improving animal handling and relocation procedures, including 42 in 2020, 157 in 2021, 145 in 2022, 121 in 2023, and 118 in 2024.

### 3.3. Escape and Recovery Dynamics

A total of 206 escape incidents were recorded, of which 204 (99.0%) involved cats and only 2 (1.0%) involved dogs.

Considering the total number of animals per species, escape rates were 0.09% (2/2254; 95% CI: 0.01–0.32%) for dogs and 8.06% (204/2531; 95% CI: 7.03–9.17%) for cats.

A statistically significant association was observed between species and escape occurrence (chi-square test, χ^2^ = 185.6, *p* < 0.0001), indicating a substantially higher likelihood of escape among cats.

Annual escape rates were 6.5% in 2020, 1.9% in 2021, 4.1% in 2022, 0.9% in 2023, and 5.45% in 2024, showing an overall reduction over time despite some variability between years.

Recovery rates differed markedly between species. While all escaped dogs were successfully recovered (100%), the recovery rate for cats was substantially lower (20.6%), highlighting a pronounced species-related disparity. This finding suggests greater challenges in locating and recovering cats once escaped.

**Figure 2:**
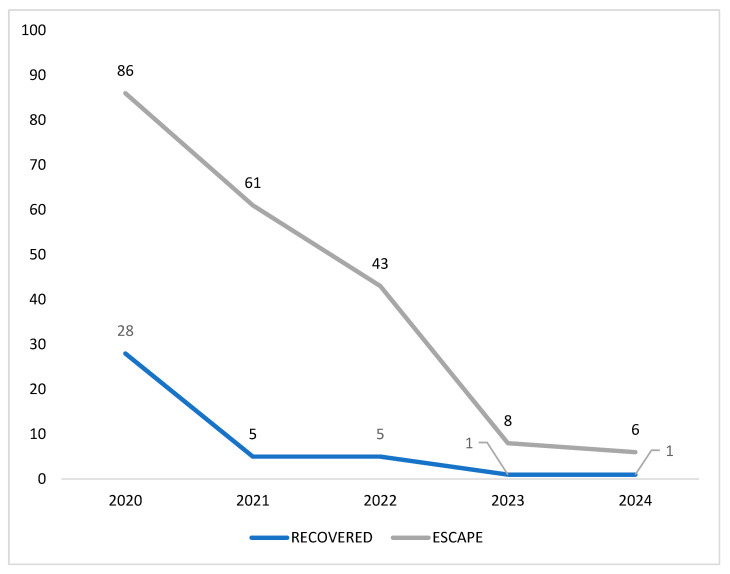
Comparison of the percentage of escapes and recovered animals during relocations in neighborhoods affected by subsidence in Maceió, Alagoas, from October 2020 to December 2024. Integra Animal Project (2024).

Annual recovery rates were 32.6% in 2020, 8.2% in 2021, 11.6% in 2022, 12.5% in 2023, and 16.7% in 2024 (Figure 2), indicating consistently lower recovery success among cats. Annual escape rates showed an overall decreasing trend over time, although some year-to-year variability was observed. This pattern may reflect progressive improvements in relocation procedures and animal management practices throughout the study period.

### 3.4. Veterinary Care and Abandonment Trends

Regarding sanitary management, 4716 animals (67.0%; *n* = 6673) received rabies vaccination and antiparasitic treatment, while 2302 (33.0%) had up-to-date vaccination records at baseline.

For population control, 873 dogs (38.7%) and 1711 cats (67.6%) were sterilized, indicating higher adherence among cat guardians.

**Figure 3:**
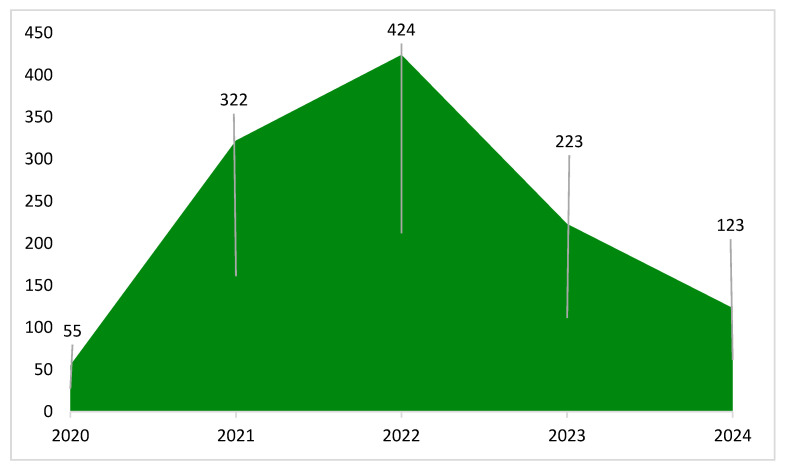
Number of animal abandonment cases recorded by the Integra Animal Project from October 2020 to December 2024. Integra Animal Project (2024).

Monitoring of abandonment cases revealed variation over time, with an overall decreasing trend throughout the study period (Figure 3), indicating a progressive reduction in recorded cases.

## 4. Discussion

The results of this study suggest that the actions implemented by the Integra Animal Project were associated with a reduction in animal abandonment and escape rates during relocations caused by land subsidence in Maceió. The increase in the number of animals registered and relocated after the implementation of the project, combined with the observed reduction in escape rates, indicates a positive trend consistent with the adoption of systematic monitoring, environmental education, and interinstitutional collaboration. These findings reinforce the importance of coordinated strategies for animal protection in socio-environmental disaster contexts, as previously reported [24,31,32].

Comparison with the pre-project scenario indicates a substantial improvement in the management of family relocation with companion animals. Between 2018 and 2020, only 43.2% of registered animals were relocated, whereas after the implementation of the project, more than 90% were effectively monitored during relocation. This shift may reflect the influence of continuous and preventive interventions, particularly those related to environmental education and responsible ownership, which are known to support behavioral change [6,28,29]. These results are consistent with studies suggesting that sustained interventions are more effective than isolated emergency responses [16,23].

Environmental education appears to have been a key component influencing the outcomes observed in this study. By promoting responsible ownership and increasing awareness of animal welfare during relocation, educational interventions may have contributed to reducing abandonment and improving animal retention [28,29,33].

Continuous engagement with guardians—through school-based activities, community outreach, and field-based guidance—likely facilitated behavioral changes, particularly in vulnerable populations facing abrupt environmental and social disruptions. Moreover, the integration of educational actions with other interventions, such as vaccination campaigns, sterilization programs, and monitoring activities, may have reinforced key messages in practical contexts, increasing adherence to recommended practices [6]. Although the study design does not allow for isolating the specific effect of education, these findings suggest that environmental education functions as an important complementary strategy within integrated approaches to animal welfare in disaster scenarios.

The marked difference in escape rates between species, supported by the chi-square analysis, indicates that cats are significantly more vulnerable to escape during relocation processes. This is further reinforced by the non-overlapping confidence intervals observed between species, highlighting a substantial disparity in risk. The predominance of cats among the monitored animals may be associated with the perception that these animals are more independent, which can lead to underestimation of risks during relocation [34].

This pattern has been discussed in the literature, where cats are often considered lower-maintenance animals, potentially influencing management practices. In addition, previous studies have reported a higher propensity for cats to escape under stress or environmental changes [35,36]. The reduction in escape events observed over time is consistent with the effects of pre-relocation preparation, professional guidance, and post-relocation follow-up, which may have contributed to improved animal handling and risk mitigation [35,36].

Health and population control actions also appear to have played a relevant role. Although 67% of animals received vaccination and antiparasitic treatment, only 33% had up-to-date vaccination records, indicating gaps in preventive care. These values are lower than estimates reported for Latin America, where approximately 60% of dogs are vaccinated, highlighting persistent inequalities in access to veterinary services [37].

Limited access to care and reliance on public campaigns remain important barriers, particularly in socioeconomically vulnerable populations, as described in previous studies [38,39]. These gaps have direct implications for both animal welfare and public health, especially in contexts with increased risk of zoonotic disease transmission [40].

Sterilization efforts were essential for population control, although resistance from guardians, particularly dog owners, remained evident. Cultural perceptions regarding sterilization continue to influence adherence, as widely reported in the literature [41]. Cultural resistance to sterilization may be influenced by misconceptions about animal health and reproduction, concerns regarding surgical risks, and traditional practices related to free-roaming animal management. Such factors may reduce adherence to population control strategies, especially in socially and economically vulnerable communities [41]. Despite this, the rates observed in this study were higher than those reported in similar programs, suggesting a gradual increase in acceptance, possibly associated with continuous educational efforts and sustained community engagement [33].

The reduction in abandonment cases over time, observed through continuous monitoring of affected areas, is consistent with the potential contribution of integrated strategies combining environmental education and active surveillance. The presence of multidisciplinary teams may have strengthened both animal care and community engagement, facilitating trust and cooperation between stakeholders. These findings align with studies highlighting the interconnectedness of animal welfare, environmental sustainability, and public health within a One Health framework [14,15,40].

Some limitations should be considered when interpreting these findings. The use of secondary data may have led to underestimation of abandonment cases, as some animals may have been removed by third parties or migrated to other areas. In addition, socioeconomic factors likely influenced adherence to recommended practices, including vaccination and sterilization. The observational design of the study, the absence of a control group, and the use of predominantly descriptive analyses limit the ability to establish causal relationships and control for potential confounding factors. Furthermore, the focus on a specific disaster context may restrict the generalizability of the results to other settings.

Despite these limitations, the strategies adopted—based on continuous monitoring, professional training, and environmental education—provide important insights for the development of integrated approaches in similar scenarios. The collaboration between public institutions, academia, and the private sector appears to be a key element in promoting effective, ethical, and sustainable responses to environmental emergencies involving companion animals.

## 5. Conclusions

This study provides field-based evidence that integrated strategies combining continuous monitoring and environmental education were associated with reduced rates of companion animal abandonment and escape during disaster-driven relocation. The higher escape rates observed among cats highlight the need for species-specific management approaches.

Although causal relationships cannot be established, the findings suggest that coordinated and sustained interventions may contribute to improved animal retention and welfare in crisis contexts. These results contribute to advancing knowledge on multispecies family relocation and may inform future strategies and public policies within a One Health framework.

## Data Availability

The data presented in this study are available on request from the corresponding author. The data are not publicly available due to privacy and ethical restrictions.

## Abbreviations

MPE-AL	Ministério Público Estadual de Alagoas
UFAL	Universidade Federal de Alagoas
CEUA	Comissão de Ética no Uso de Animais
